# The Innovation Imperative: Time to Rewrite Academia’s Compact With Industry

**DOI:** 10.7759/cureus.84103

**Published:** 2025-05-14

**Authors:** Shaheen E Lakhan

**Affiliations:** 1 Medical Office, Click Therapeutics, New York, USA; 2 Neurology, Western University of Health Sciences, Pomona, USA; 3 Neurology, A.T. Still University School of Osteopathic Medicine in Arizona, Mesa, USA; 4 Neurology, Morehouse School of Medicine, Atlanta, USA; 5 Bioscience, Global Neuroscience Initiative Foundation, Miami, USA

**Keywords:** academic medicine, academic metrics, commercialization, entrepreneurial academia, faculty promotion, industry partnerships, innovation ecosystems, public-private collaboration, tech transfer reform, translational science

## Abstract

Academic medicine is at a crossroads. Amid historic federal disinvestment in research and persistent cultural resistance to industry collaboration, the traditional academic compact, based on grants, publications, and tenure, is no longer sustainable. Drawing on firsthand experience as a medical school dean, faculty, and biotech executive, this editorial argues that academic institutions must urgently reform how they value and support entrepreneurial scholarship. With fewer than 1% of university patents leading to licensed products, the prevailing model of late-stage, passive tech transfer has failed to translate innovation into impact. Meanwhile, global models from the Israel Innovation Authority to Singapore’s Biopolis demonstrate the power of early, structured academia-industry partnerships. This piece proposes a new framework for evaluating faculty achievement, one that elevates patents, startups, regulatory wins, clinical product launches, and translational teaching as core academic contributions. It calls for institutional reforms to faculty appointment and promotion criteria, dedicated innovation offices, and policy incentives to reward real-world impact. The innovation imperative is clear: if academic medicine is to remain relevant, it must become an engine not only of knowledge, but of change.

## Editorial

After decades in academic medicine, as a faculty member, department chair, founding dean, and author of faculty bylaws, and later as a biotech executive and board director, I have witnessed the immense promise and persistent inertia of academic research. Our universities are home to brilliant minds and groundbreaking discoveries. Yet, too often these innovations remain confined to lab notebooks, manuscripts, or conference slides, never reaching the patients they were meant to help.

At the same time, federal funding, the traditional lifeline for academic research, is drying up and drying up fast. Intensified under the Trump administration with historically deep cuts to scientific research [1], budgets at the National Institutes of Health (NIH) and National Science Foundation (NSF) are being slashed. These dramatic reductions have triggered national alarm. Many scientists from the National Academies of Sciences, Engineering, and Medicine have issued an open letter warning of irreversible damage to America’s research ecosystem [2].

But within this crisis lies a generational opportunity: to finally shed the outdated norms that valorize only publications, grants, and h-indexes, and instead embrace a broader, bolder vision of academic impact, one that includes entrepreneurship, translational science, and deep partnerships with industry. To get there, we must flip long-held cultural scripts, update institutional incentives, and reimagine what academic success really means in the 21st century.

The old compact is broken

For much of the 20th century, academic research thrived on a stable, singular engine: federal grants. The post-World War II surge in public investment, epitomized by the creation and expansion of the NIH, created a golden era of basic science, rooted in the belief that discovery would naturally trickle down into translation. And for a time, this worked. Universities became intellectual hubs, churning out new theories, mechanisms, and even molecular targets that seeded future therapies.

Over time, this model ossified. Academic success became defined almost exclusively by metrics tethered to grant dollars, impact factors, and citation counts. Meanwhile, "tech transfer" emerged as academia’s perfunctory nod to commercialization: invent a thing, patent it, hand it off to a licensing office, and hope someone in industry bites. But most never did. The vast majority of university-held patents languish. Fewer than 1% lead to licensed, marketable products, and even fewer make it to patients [3].

This handoff model, linear, transactional, and late-stage, is fundamentally misaligned with how real-world innovation happens. It assumes translation is someone else’s job. But in today’s ecosystem, where timelines are compressed and risks are high, passive licensing is no longer viable. Discovery must be paired with delivery from the outset. Academia must stop outsourcing impact.

The federal pullback and its fallout

Even if the old model worked better than it does today, it is increasingly unsustainable. Federal research dollars, the backbone of academic inquiry, have not kept up with the cost of doing science. The average age has risen above 43 years for a first-time principal investigator on an R01, meaning that most scientists do not receive their first major, independent federal research grant until their early-to-mid 40s [4]. Paylines at leading NIH institutes are at record lows. Seed grants have shriveled. Bridge funding is elusive. Institutions now rely more on soft money, further precarizing the careers of junior and mid-career faculty.

The consequences are dire. Talented early-career investigators are abandoning academic research, discouraged by the odds and instability. Senior scientists spend more time chasing funding than mentoring or innovating. The system rewards grant writing more than risk-taking, leading to fewer moonshots and more incrementalism.

In my experience overseeing departments and launching medical schools, I have seen the toll this takes firsthand. Faculty burnout is real, not because the work is hard, but because the system is brittle. We ask our best scientists to be grant accountants, paper machines, and endless committee members, while giving them little support to translate their insights into scalable impact.

Industry as partner, not pariah

Despite mounting pressures, many in academia still view industry partnerships with skepticism or outright disdain. Industry is seen as impure, commercial, biased, and driven by profit rather than principle. In some institutions, faculty may face discouragement or even be penalized for engaging in consulting, advisory roles, or entrepreneurial ventures. Industry affiliations are too often whispered, disclosed defensively, or omitted altogether from CVs.

This cultural taboo is both outdated and dangerous. In the real world, industry is not the enemy of innovation; it is its engine. Industry offers not just funding but also executional capacity, regulatory expertise, market validation, and speed. The dichotomy of “pure science” vs. “applied science” has always been a false one. Some of the most important medical breakthroughs of our time have come from codeveloped programs: messenger RNA vaccines, oncology diagnostics, neuromodulation devices, and now, prescription digital therapeutics [5].

As someone who now serves on biotech boards and leads digital health innovation, I know firsthand how hungry the private sector is for authentic academic partnership. However, the structures, both cultural and operational, within universities often repel rather than attract such engagement. We need to build a new, mutually respectful language of collaboration that starts early, shares risk, and measures success not by publication volume, but by human impact.

Models that work: global and domestic

The good news is that better models already exist. Israel, where I lived, trained, and practiced for years, offers one of the world’s most successful examples of academia-industry-government integration. The Israeli innovation ecosystem does not wait for tech transfer; it builds translational thinking into the DNA of its institutions. The Israel Innovation Authority coordinates early-stage risk capital, applied research programs, and industry matchmaking. Universities like my alma mater, The Technion - Israel Institute of Technology, and the Weizmann Institute, routinely codevelop technologies with commercialization in mind from day one.

This is not accidental. It is structural. Israeli academia accepts that impact is not a dilution of scholarship, but its extension. Researchers are celebrated, not punished, for launching companies, securing venture capital, and working with industry to ensure scientific discoveries become societal benefits.

Singapore provides another example: Biopolis and the Agency for Science, Technology and Research initiative show how entire innovation districts can be organized around seamless academic-industry collaboration. Europe’s Innovative Medicines Initiative and the more recent Innovative Health Initiative offer competitive, co-funded mechanisms that bring academia and industry into aligned, milestone-driven partnerships.

In the US, institutions like the University of California, San Francisco, with its Catalyst Program, or Stanford’s SPARK model, embed entrepreneurial thinking upstream, not as an afterthought but as a foundational expectation. The lesson is clear: when academia treats industry as a codeveloper, rather than a post hoc licensee, innovation flourishes.

Fixing the faculty compact: elevating entrepreneurial academics

One of the most urgent reforms needed is within academia itself: we must change how we value, support, and promote entrepreneurial academics. I have written faculty bylaws, chaired appointment and promotion committees, and helped design institutional handbooks. I know how rigid and outdated these documents can be. They emphasize grants secured, papers published, and students mentored, but rarely reward patents, company formation, licensing revenue, or clinical impact.

This must change. Innovation is scholarship. Translation is teaching. A successful startup, a breakthrough designation, or a Food and Drug Administration-approved therapy should count just as much as a New England Journal of Medicine article, if not more.

Universities should revise tenure criteria to include commercialization activities and strategic partnerships, establish innovation tracks that provide equal prestige and permanence to research and clinical tracks, create formal rubrics that equate measurable real-world impact with traditional academic outputs, offer protected time and support for entrepreneurial faculty, including access to seed capital and external advisors, and normalize public-private collaboration as a form of modern academic service. Table 1 summarizes reforms needed to elevate entrepreneurial contributions as core academic achievements.

**Table 1:** Recalibrating academic metrics to elevate entrepreneurial faculty This author-generated table contrasts traditional academic achievements with entrepreneurial contributions and offers reform recommendations to integrate innovation into faculty evaluation systems. It proposes a new compact in which activities such as company formation, IP generation, regulatory achievements, and strategic partnerships are formally recognized as scholarly outputs equivalent in value to grants, publications, and teaching. This framework supports the institutionalization of translational science and entrepreneurial leadership within academia Without these reforms, we risk losing our most creative faculty, those who straddle the boundary between lab and market, not because they fail, but because they do not conform to outdated metrics of success IP: intellectual property; FDA: Food Drug Administration

Traditional academic metric	Entrepreneurial academic contribution	Reform recommendation
Peer-reviewed publications	Patents filed/provisioned/granted, IP portfolio developed	Equate patents and peer-reviewed publications in appointment and promotion criteria
Grant funding secured	Startup company formation, venture funding raised	Treat successful startups and funding as research impact
Teaching and mentoring	Translational education, regulatory mentorship	Include translational teaching and entrepreneurial mentorship in evaluations
Professional service (e.g., committees)	Public-private partnerships, industry consortia participation	Acknowledge strategic partnerships as academic service
Conference presentations	FDA authorizations, designations, and clinical product launches	Reward regulatory and commercialization outcomes as high-impact scholarship

Without these reforms, we risk losing our most creative faculty, those who straddle the boundary between lab and market, not because they fail, but because they do not conform to outdated metrics of success.

Building new bridges

To operationalize this shift, academic institutions must build the infrastructure for sustained, strategic partnerships. This means going beyond tech transfer offices to establish dedicated units for external engagement, offices of innovation, entrepreneurship, and strategic partnerships. These teams should speak the languages of both science and business, helping faculty navigate regulatory pathways, pitch decks, intellectual property strategy, and reimbursement models.

University leadership must take a stand: encourage faculty to participate in industry consortia, accept equity as compensation where appropriate, and build sabbatical programs that allow time in startups or biotech firms.

Policy reform can help. The Internal Revenue Service should offer tax incentives for joint research and development. Federal agencies could pilot cosponsored grant mechanisms with industry (e.g., hybrid NIH/Small Business Innovation Research programs). Venture philanthropy should be encouraged as a vehicle to support high-risk academic innovations with social return.

More broadly, we must create an academic climate that celebrates, not penalizes, collaborative impact. If translational science is truly our mission, we must rewrite the social contract to reward those who make it real.

Conclusion: rewriting the academic social contract

We are standing at a crossroads. The Trump administration’s cuts and further proposed cuts to NIH and NSF are not just budgetary events; they are clarifying moments. They expose the fragility of a research enterprise that is too dependent on federal largesse and too slow to adapt to a changing world.

But this is not a crisis of loss. It is a crisis of opportunity. Academia can remain relevant, indispensable even, if it evolves. That means rethinking how we define scholarship. It means tearing down the walls that separate discovery from delivery. And it means empowering a new generation of scientists who want to not only understand disease but to defeat it.

I have lived this transformation, from the classroom to the clinic, from the bench to the boardroom. I believe that with the right reforms, our universities can become not just engines of knowledge, but engines of change. It is time to build a new era of academic medicine, one defined not by intention but by impact.
